# Secondary Formation of Aromatic Nitroderivatives of Environmental Concern: Photonitration Processes Triggered by the Photolysis of Nitrate and Nitrite Ions in Aqueous Solution

**DOI:** 10.3390/molecules26092550

**Published:** 2021-04-27

**Authors:** Giovanna Marussi, Davide Vione

**Affiliations:** 1 Dipartimento di Scienze Chimiche e Farmaceutiche, Università degli Studi di Trieste, Via Licio Giorgieri 1, 34127 Trieste, Italy; giovanna.marussi@phd.units.it; 2 Dipartimento di Chimica, Università degli Studi di Torino, Via Pietro Giuria 5, 10125 Torino, Italy

**Keywords:** nitroaromatic compounds, nitrophenols, atmospheric chemistry, environmental photochemistry, secondary contaminants, photogenerated nitrating agents

## Abstract

Aromatic nitroderivatives are compounds of considerable environmental concern, because some of them are phytotoxic (especially the nitrophenols, and particularly 2,4-dinitrophenol), others are mutagenic and potentially carcinogenic (e.g., the nitroderivatives of polycyclic aromatic hydrocarbons, such as 1-nitropyrene), and all of them absorb sunlight as components of the brown carbon. The latter has the potential to affect the climatic feedback of atmospheric aerosols. Most nitroderivatives are secondarily formed in the environment and, among their possible formation processes, photonitration upon irradiation of nitrate or nitrite is an important pathway that has periodically gained considerable attention. However, photonitration triggered by nitrate and nitrite is a very complex process, because the two ionic species under irradiation produce a wide range of nitrating agents (such as ^•^NO_2_, HNO_2_, HOONO, and H_2_OONO^+^), which are affected by pH and the presence of organic compounds and, in turn, deeply affect the nitration of aromatic precursors. Moreover, aromatic substrates can highly differ in their reactivity towards the various photogenerated species, thereby providing different behaviours towards photonitration. Despite the high complexity, it is possible to rationalise the different photonitration pathways in a coherent framework. In this context, this review paper has the goal of providing the reader with a guide on what to expect from the photonitration process under different conditions, how to study it, and how to determine which pathway(s) are prevailing in the formation of the observed nitroderivatives.

## 1. Introduction

The photonitration process is the formation of nitroderivatives from suitable substrates under irradiation conditions [1]. Irradiation is usually essential to the formation of the reactive species (nitrating agents), but in some cases it may also play a key role in activating the substrate to the nitration reaction, which would otherwise not take place in the dark with the same nitrating agent (vide infra for the case of the nitration of nitrophenols) [2].

Starting from earlier works in the early 1980s [3,4], studies on photonitration in aqueous solution have focused on processes that are triggered by the photochemistry of nitrate and nitrite ions. The fact that the reaction takes place in the aqueous phase, the wide environmental occurrence of both nitrate and nitrite, as well as the ability of both ions to absorb environmental UV radiation from sunlight [5], explain why the photonitration reactions have attracted interest, in an attempt to account for the formation of nitroderivatives in surface waters and, most notably, in atmospheric waters and aerosols.

In particular, there has been interest towards photonitration in three successive periods—(i) from the early 1980s to the mid-1990s, when the main goal was to account for the formation of phytotoxic nitrophenols that, together with acid rains, play a role in forest decline [6,7]; (ii) the 2000s, when the process attracted attention to account for the occurrence (and secondary formation) of toxic, mutagenic and potentially carcinogenic nitroaromatic compounds in atmospheric aerosols [8,9]; and (iii) the present time, when most interest is accounted for by the fact that the –NO_2_ group of nitroderivatives enables them to absorb sunlight (visible radiation in addition to the UV, because most nitroderivatives are yellow both as solids and in aqueous solution). As part of the so-called “brown carbon”, nitroaromatic compounds play a role in the absorption of sunlight by airborne aerosols and, by so doing, they affect to a potentially significant extent the still-debated and insufficiently defined climate feedback of the aerosols [10,11,12,13,14,15,16]. Another reason for the recent interest towards photonitration is the acknowledgement of the important role it may play in photochemical processes for water treatment, which are gaining increasing attention and where the occurrence of nitrate and nitrite under technical UV irradiation might favour the formation of harmful compounds [17,18,19,20,21].

Whatever the reason for the focus on the photonitration reactions, most of the relevant studies had/have to address the issue of the photonitration pathway(s). On the one side, this task is apparently made easy by the fact that both nitrate and nitrite are able to produce the nitrating agent ^•^NO_2_ (nitrogen dioxide) under irradiation [22]. However, the photonitration process has several features (effect of pH, because photonitration is usually enhanced under acidic conditions, and effect of the addition of scavengers of the hydroxyl radical, ^•^OH, which are very common in the natural environment) that often have to be explained as well. Actually, the attempts to account for the whole features of the photonitration process, based on the role of ^•^NO_2_ as the sole nitrating agent, and on the pH effect as only affecting the production of ^•^NO_2_ by nitrate and nitrite have proven, on the whole, largely unsatisfactory [1]. To make things worse, photonitration appeared to induce the formation of nitroderivatives from substrates that were too electron-poor to react with ^•^NO_2_ [2]. Indeed, more detailed studies have shown that irradiation of both nitrate and nitrite produces a plethora of potential nitrating agents [23,24], in different amounts (also depending on the conditions, e.g., the pH of the solution) and with different reactivity, which interact with each substrate to finally produce the observed effects. Therefore, the photonitration process is not amenable to a simple explanation, based on the role of a single nitrating agent.

Unfortunately, essential information about the photonitration pathway(s) has been fragmented in several different publications, and there is currently not a single paper where all the mechanistic findings are summarised and compared. This review paper is intended to fill this gap, also in view of the recent interest towards the photochemical formation of nitroderivatives as brown-carbon components, and as potential contaminants of treated water and wastewater. Therefore, researchers working in the photonitration field could find here a reference and a guide through an environmentally interesting but, at the same time, very tricky process. This review paper deals with the photonitration of aromatic compounds, which has been the main research focus so far. However, it is important to point out that the formation of nitro- and nitrosoderivatives under irradiation has also been observed in the presence of aliphatic precursors such as primary amines [25], which have the potential to yield toxic and carcinogenic compounds.

The present review paper starts by describing the processes that follow the irradiation of both nitrate and nitrite ions, which on the one side are long known [22], but where some key details have been fully elucidated only more recently, to account for some features of the photonitration process [23,26]. It follows an account of the photonitration of phenol, a substrate that can react with virtually all photogenerated nitrating agents—the elucidation of phenol photonitration pathways was actually the starting point to really understand the nature of the process [27]. Peculiar case studies are then presented, with substrates that help highlight particular reactions, and for which the photonitration mechanism has been convincingly elucidated within a coherent framework. Finally, considering that there is quite convincing evidence for aromatic photonitration to occur in natural surface waters [5,28,29,30], at least under particular conditions [31], the details of such a process are shown as well. Because natural waters are much more complex than laboratory model systems, allowance is here made for the likely interaction between different surface-water components. Conclusions follow in the end.

## 2. Aromatic Photonitration in Aqueous Solution

### 2.1. Nitrate (NO_3_^−^) Photolysis

Nitrate absorbs sunlight up to approximately 340 nm. It has a weak absorption maximum at 302 nm (ε_max_ = 7.2 L mol^−1^ cm^−1^) and, because the irradiance of sunlight increases with increasing wavelength in the UV region, one gets that sunlight absorption by nitrate is most effective at around 315–320 nm. Radiation absorption breaks the NO_3_^−^ ion into two fragments, namely O^•−^ and ^•^NO_2_, where O^•−^ is the conjugate base of the hydroxyl radical, ^•^OH [22,32]:

NO_3_^−^ + hν → [^•^NO_2_ + O^•^^−^]_cage_  [Φ = 0.01] (1)

where Φ is the quantum yield of nitrate photolysis (ratio between photolysed molecules and absorbed photons). The two photogenerated fragments are initially located inside a cage of water molecules, where three alternative processes can take place [23,33]:

1. Diffusion of ^•^NO_2_ + O^•−^ out of the solvent cage and into the solution bulk, where O^•−^ undergoes prompt protonation to ^•^OH if pH < 12;
2. Geminate recombination of the two fragments back to the original NO_3_^−^; and
3. Recombination of the fragments to form peroxynitrite, ONOO^−^, which is an isomer of nitrate (this latter process takes the form of nitrate photoisomerisation). Peroxynitrite is the conjugate base of the weak acid HOONO (pK_a_ = 7), peroxynitrous acid. HOONO is an unstable species that can either give back nitrate or decompose into ^•^OH + ^•^NO_2_. In contrast, OONO^−^ mainly reacts with dissolved CO_2_ [24].

[^•^NO_2_ + O^•−^]_cage_ → ^•^NO_2_ + O^•−^ (2)

O^•^^−^ + H^+^ ⇆ ^•^OH  [pK_a_(^•^OH) = 11.9] (3)

[^•^NO_2_ + O^•−^]_cage_ → NO_3_^−^ (4)

[^•^NO_2_ + O^•−^]_cage_ → OONO^−^ (5)

OONO^−^ + CO_2_ → ONOOCO_2_^−^ (6)

OONO^−^ + H^+^ ⇆ HOONO (7)

HOONO → NO_3_^−^ (8)

HOONO → ^•^OH + ^•^NO_2_ (9)

**Scheme 1:**
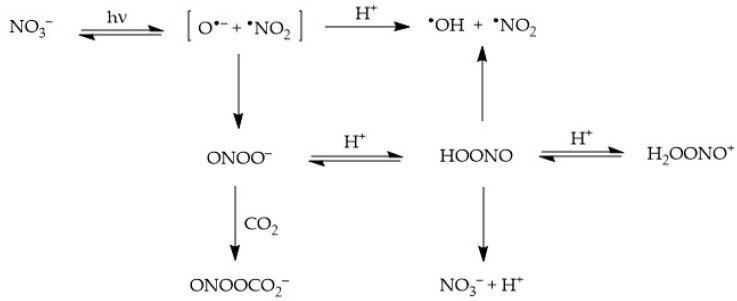
Primary photoprocesses and early subsequent reactions triggered by the photolysis of NO_3_^−^.

The processes triggered by nitrate photolysis are summarised in the following Scheme 1.

Among the species formed by nitrate photolysis, ^•^NO_2_, HOONO and H_2_OONO^+^ (the conjugate acid of HOONO; note that H_2_OONO^+^ has pK_a_ < 0) are all nitrating agents [2,34]. Nitrogen dioxide is unstable in aqueous solution, where it undergoes dimerisation to N_2_O_4_ and hydrolysis [22,35,36]:

2 ^•^NO_2_ ⇆ N_2_O_4_ [*k* = 4.5 × 10^8^ L mol^−1^ s^−1^; *k*_−_ = 6.9 × 10^3^ s^−1^] (10)

N_2_O_4_ + H_2_O → HNO_2_ + NO_3_^−^ + H^+^ [k = 1.0 × 10^3^ s^−1^] (11)

HNO_2_ ⇆ H^+^ + NO_2_^−^ [pK_a_ = 3.3] (12)

Nitrous acid, HNO_2_, is another nitrating agent that is active towards electron-rich compounds such as phenols [14].

### 2.2. Nitrite (NO_2_^−^) Photolysis

The nitrite anion has two main absorption bands in the environmental UV region. The lower-wavelength band appears as a 270–280 nm shoulder in the nitrite absorption spectrum, and it extends into the UVB region where it overlaps with the higher-wavelength band. The latter produces a clear absorption maximum at around 365 nm. Radiation absorption by nitrite in the environmental UV region (both UVB and UVA) causes photolysis with generation of ^•^NO + O^•−^ as the primary process, and of ^•^NO_2_ + e^−^_aq_ as a secondary one [22]:

NO_2_^−^ + hν → ^•^NO + O^•−^ [Φ’ = 0.025–0.065] (13)

O^•−^ + H^+^ ⇆ ^•^OH (14)

NO_2_^−^ + hν → ^•^NO_2_ + e^−^_aq_ [Φ″ ~ 0.001] (15)

The quantum yield Φ′ of Reaction (13) depends on the nitrite band that is involved in radiation absorption. Indeed, the short-wavelength band has a quantum yield Φ’ = 0.065, while the long-wavelength band has Φ′ = 0.025. In the environmental UV range, absorption by NO_2_^−^ in the short-wavelength band decreases as the wavelength increases, while absorption in the long-wavelength band does the reverse, at least up to the maximum at 365 nm. As a consequence, the quantum yield Φ′ has a value of 0.065 at 280–290 nm, which decreases down to 0.025 in the UVA region [22]. Interestingly, nitrite can be oxidised by the same ^•^OH radicals it generates under irradiation, with subsequent production of ^•^NO_2_:

NO_2_^−^ + ^•^OH → ^•^NO_2_ + OH^−^ [*k* = 1.0 × 10^10^ L mol^−1^ s^−1^] (16)

The above reaction accounts for most of the formation of the nitrating agent ^•^NO_2_ in the presence of irradiated nitrite [27,37]. Interestingly, the occurrence of ^•^NO and ^•^NO_2_ in the same system triggers an efficient process of nitrite regeneration:

^•^NO + ^•^NO_2_ ⇆ N_2_O_3_ [*k* = 1.1 × 10^9^ L mol^−1^ s^−1^] (17)

N_2_O_3_ + H_2_O → 2 NO_2_^−^ + 2 H^+^ [*k* = 5.3 × 10^2^ s^−1^] (18)

**Scheme 2:**
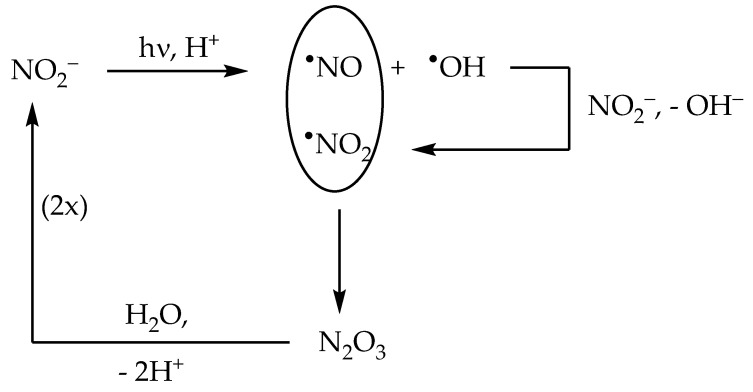
Primary photoprocesses and early subsequent reactions triggered by the photolysis of NO_2_^−^.

Therefore, although NO_2_^−^ is oxidised by ^•^OH, this reaction plays a key role in the regeneration of NO_2_^−^ rather than in its degradation. As a consequence, the disappearance of NO_2_^−^ is considerably faster in the presence of ^•^OH scavengers that inhibit Reaction (16) and, therefore, inhibit nitrite regeneration as well [38]. The processes described above are summarised in Scheme 2.

Nitrite is a weak base that forms nitrous acid in acidic solution (HNO_2_, also indicated as HONO in the field of atmospheric chemistry). HNO_2_ is an unstable weak acid with pK_a_ = 3–3.5; it absorbs radiation in about the same wavelength range as nitrite, and with roughly comparable absorption coefficients (λ_max_ = 371 nm, ε_max_ = 51.9 L mol^−1^ cm^−1^). The photolysis of nitrous acid yields ^•^NO, ^•^OH and ^•^NO_2_ in a similar way as nitrite photolysis [22,39]:

HNO_2_ + hν → ^•^NO + ^•^OH [Φ = 0.35] (19)

HNO_2_ + ^•^OH → ^•^NO_2_ + H_2_O [*k* = 2.6 × 10^9^ L mol^−1^ s^−1^] (20)

However, the direct photolysis quantum yield of HONO (Φ = 0.35) is much higher than that of nitrite (Φ = 0.025–0.065), thus the photochemical reactivity of nitrite-containing solutions increases as the pH decreases. Interestingly, the UVA absorption spectrum of HONO shows a vibrational fine structure that is lacking in the nitrite spectrum. Moreover, HONO absorption is not extended into the visible region, while concentrated nitrite solutions are pale yellow.

**Figure 1:**
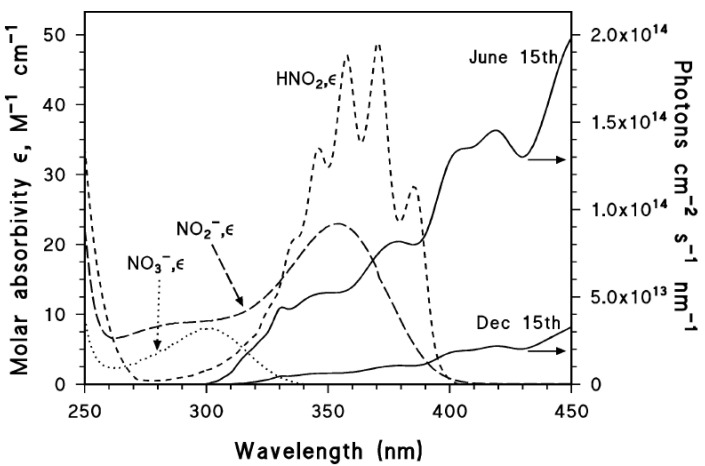
**Left y axis.** Molar absorption coefficients of NO_3_^−^, NO_2_^−^ and HNO_2_. **Right y axis.** Spectral photon flux density of sunlight in mid-latitude conditions, during summertime and wintertime (northern hemisphere). Vione, unpublished data.

The absorption spectra of nitrate, nitrite and nitrous acid are reported in Figure 1, where they are compared with summertime and wintertime spectra of sunlight under mid-latitude conditions.

### 2.3. A Brief Overview of the Different Photogenerated Nitrating Agents

As shown in the previous sections, the irradiation of nitrate and nitrite yields different nitrating agents such as ^•^NO_2_, HNO_2_, and HOONO (which can be partially protonated to H_2_OONO^+^ in strongly acidic conditions). Electron-rich aromatics will mostly be nitrated by ^•^NO_2_ or HNO_2_ that are formed or occur in a higher amount [6,26], while HOONO/H_2_OONO^+^ (however powerful their potential towards aromatic nitration may be [40,41,42]) are produced with low quantum yield by nitrate irradiation. Therefore, the reactivity of HOONO/H_2_OONO^+^ will only be highlighted in the presence of deactivated aromatic compounds that do not react with ^•^NO_2_/HNO_2_ [2].

**Figure 2:**
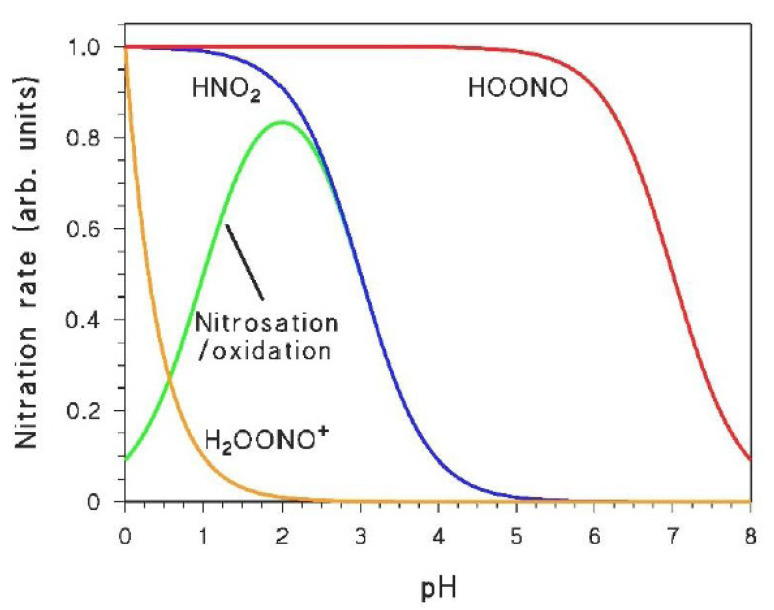
Trends, as a function of pH, of the occurrence of nitrating agents (or of nitration processes) that are produced (or can be triggered) under irradiation.

The role of the different nitrating agents in the photonitration of a given compound can be highlighted when taking into account the peculiar pH trends that characterise at least some of them. Indeed, H_2_OONO^+^ has pK_a_ < 0, HOONO has pK_a_ ~ 7, while HNO_2_ has pK_a_ ~ 3.5 [22,40]. Moreover, HNO_2_ can also be involved in a nitrosation/oxidation process that gives a maximum in nitroaromatic formation at pH 2–3 [43]. Figure 2 shows the pH trends that characterise the occurrence and/or reactivity of the different nitrating agents, and that may be reflected in the rates of nitroaromatic formation as a function of pH. Indeed, a study of the pH trend should be a key step when trying to understand the aromatic photonitration pathways (vide infra).

The following sections provide a number of case studies concerning the photonitration of several aromatic compounds, highlighting the main nitrating agents that are involved in the process and, when available, describing the underlying nitration mechanism(s).

### 2.4. Phenol Photonitration Pathways

**Scheme 3:**
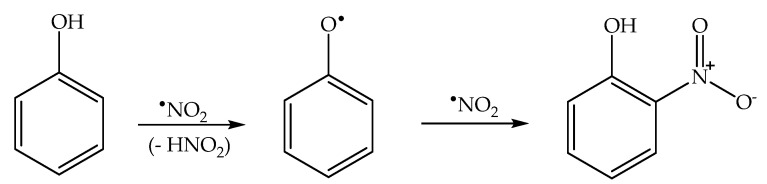
Nitration pathway of phenol by ^•^NO_2_. The formation of 2NP and 4NP follow similar pathways.

Formation of the ortho and para nitrophenol isomers (2-nitrophenol and 4-nitrophenol, hereinafter respectively 2NP and 4NP) from phenol has been observed upon irradiation of both nitrate and nitrite [1,6]. In neutral solution, in both cases, the photonitration process has been shown to involve a reaction between phenol and ^•^NO_2_, with the likely involvement of the phenoxyl radical as a reaction intermediate (Scheme 3). This most likely reaction pathway has been suggested by quantum chemistry calculations as well [27].

It is interesting to observe that the formation of 2- and 4-nitrophenol is peculiar for the nitration of phenol, which does not yield the meta isomer (3-nitrophenol). In contrast, formation of the latter compound (together with the other two nitrophenol isomers) has been observed upon hydroxylation of nitrobenzene [44].

The photonitration of phenol upon irradiation of nitrate has three main features, the explanation of which has been key to understanding the reaction pathway(s) involved—(i) it is favoured in the absence of oxygen; (ii) it is enhanced upon addition of ^•^OH scavengers such as 2-propanol; (iii) it is enhanced under acidic pH conditions [1,6].

**Scheme 4:**
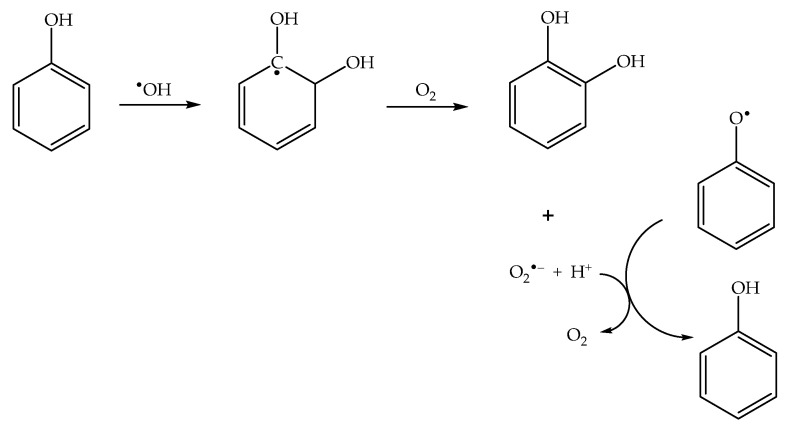
Photoinduced formation of superoxide, and its role in phenoxyl reduction to phenol.

In the presence of dissolved oxygen, different reaction pathways yield the superoxide radical anion O_2_^•^^−^ (indeed, HO_2_^•^/O_2_^•^^−^ are typical products of the reactions between organics and ^•^OH [45]). Superoxide is able to reduce phenoxyl back to phenol [46] (Scheme 4), thereby inhibiting the photonitration process. Therefore, nitrophenol formation is enhanced in the absence of oxygen.

**Scheme 5:**
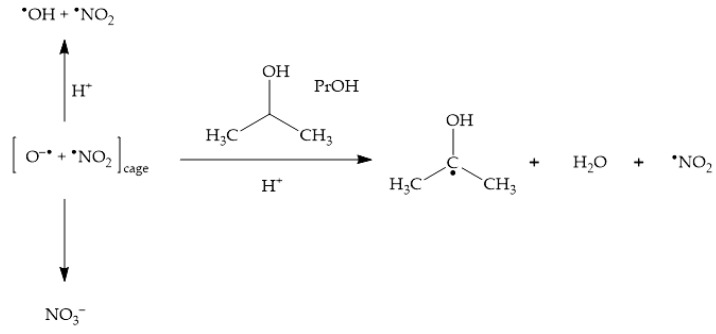
Enhancement of ^•^NO_2_ formation upon nitrate photolysis by the ^•^OH scavenger 2-propanol (PrOH).

The addition of ^•^OH scavengers at high enough concentration leads to the consumption of not only bulk ^•^OH, but also of cage O^•−^ [23,47]. By inhibiting cage recombination between O^•−^ and ^•^NO_2_, scavengers such as 2-propanol (PrOH) enhance the photochemical production of ^•^NO_2_ (Scheme 5), thereby enhancing as well the photonitration process [23].

**Scheme 6:**
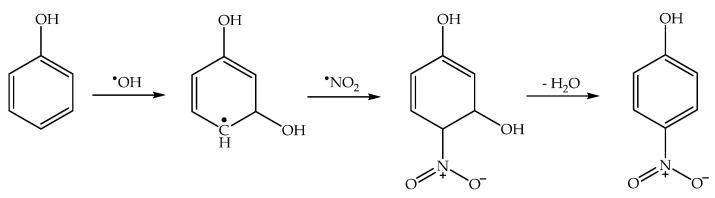
Phenol nitration by ^•^OH + ^•^NO_2_, which is not operational as photonitration pathway.

**Scheme 7:**
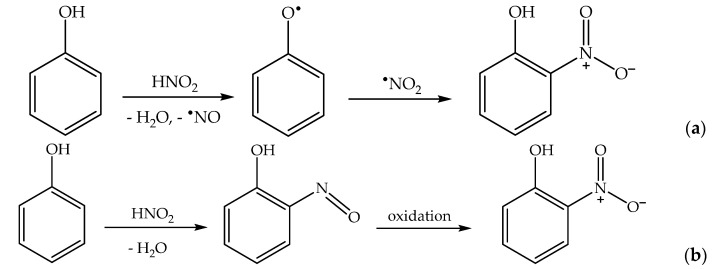
Phenol nitration pathways by HNO_2_: (**a**) direct nitration; (**b**) nitrosation/oxidation. Note that similar processes would produce 4NP in addition to 2NP.

Interestingly, the enhancement of photonitration by ^•^OH scavengers allows for the exclusion of a photonitration pathway involving ^•^OH + ^•^NO_2_ (Scheme 6), proposed in analogy with some gas-phase nitration processes [48,49]:

Finally, under acidic pH conditions, one has the production of HNO_2_ upon ^•^NO_2_ hydrolysis (reactions 10,11), and HNO_2_ is another effective nitrating agent for phenols [50]. In particular, the nitration of phenol by HNO_2_ follows two reaction pathways—a direct one, where HNO_2_ is initially involved in the oxidation of phenol to phenoxyl, and a nitrosation/oxidation process (Scheme 7). Nitration by HNO_2_ alone is enabled by the decomposition of HNO_2_ into ^•^NO + ^•^NO_2_ [43,51].

2 HNO_2_ → ^•^NO + ^•^NO_2_ + H_2_O (21)

The involvement of HNO_2_ in phenol photonitration with nitrate in acidic solution is supported by the fact that the initial formation rates of 2NP and 4NP both show an inflection point at pH ~ 3.5, which is fully compatible with the pK_a_ of HNO_2_ [52].

**Scheme 8:**
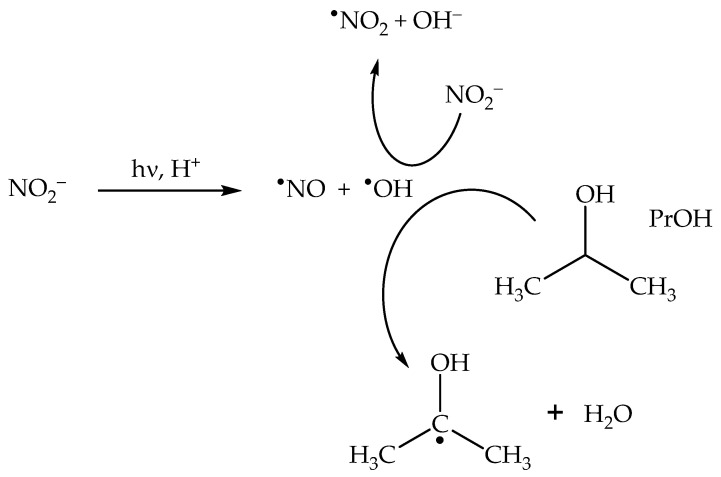
Inhibition of ^•^NO_2_ formation upon nitrite photolysis by the ^•^OH scavenger 2-propanol (PrOH).

In the presence of irradiated nitrite, the formation rate of nitrophenols is considerably higher compared to the case of nitrate. The photonitration of nitrophenols by nitrite has the following features—(i) it is inhibited by the addition of ^•^OH scavengers, and (ii) it is enhanced at acidic pH [6]. The addition of ^•^OH scavengers such as PrOH inhibits the oxidation of NO_2_^−^ to ^•^NO_2_ by ^•^OH (Scheme 8), thereby inhibiting phenol nitration as well [6,52].

The pH effect in the presence of nitrite shows the same inflection point at pH ~ 3.5 already seen with nitrate, which strongly suggests the involvement of HNO_2_. However, in acidified nitrite solutions both the thermal process (direct reaction between phenol and HNO_2_, or nitrosation/oxidation) and the photoinduced one (higher photoreactivity of HNO_2_ compared to NO_2_^−^ to yield ^•^NO_2_) are operational at the same time. Therefore, with NO_2_^−^ at pH < 5 it is very difficult to understand which fraction of nitrophenol formation is thermal and which is photoinduced, although experimental evidence points at a prevalence of the thermal process [53].

To sum up, phenol as an electron-rich aromatic compound can undergo photonitration by virtually all the nitrating species generated by nitrate and nitrite under irradiation—^•^NO_2_, HNO_2_, HOONO and H_2_OONO^+^. However, the roles of ^•^NO_2_ and HNO_2_ strongly prevail over those of HOONO and H_2_OONO^+^, which cannot be highlighted to a significant extent in the presence of important competing processes. In contrast, nitrophenol formation has been reported in the presence of HOONO, thermally produced upon the reaction between HNO_2_ and H_2_O_2_ [54], and robust evidence confirms the ability of HOONO to nitrate phenolic compounds [55]. The issue with photonitration is different, because HOONO and H_2_OONO^+^ are generated in quite a small amount.

It is also very interesting to highlight that both ^•^NO_2_ and HNO_2_ are able to oxidise phenol to the phenoxyl radical [16,27,43], which is a key step in the (photo)nitration process.

### 2.5. The Photonitration of Nitrophenols

Phenol is amenable to photonitration as shown in the previous section, but this activated aromatic compound can be nitrated under a very wide variety of conditions, both thermal (which in atmospheric waters might also involve N_2_O_5_, produced by reaction of ^•^NO_3_ + ^•^NO_2_, in addition to HNO_2_), and photochemical [56]. Therefore, secondary environmental nitrophenols might in principle arise from a plethora of nitration pathways, of which photonitration is only one of the possibilities. In contrast, it is quite difficult to account for the nitration of nitrophenols (2NP, 4NP) to 2,4-dinitrophenol (24DNP), i.e., the most phytotoxic nitrophenol compound [56,57], without invoking the photoinduced nitration pathways.

**Scheme 9:**
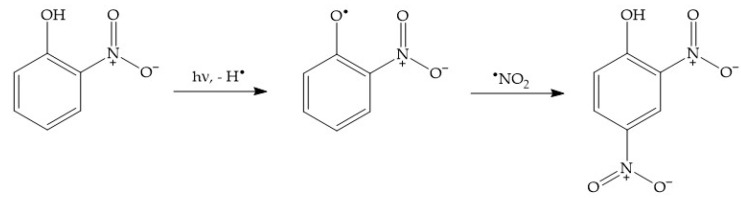
Photonitration pathway of 2NP to 24DNP. Note that a similar process would involve 4NP, to also produce 24DNP.

For instance, dark nitration of nitrophenols by ^•^NO_2_ or HNO_2_ can be excluded, because neither reactant is able to oxidise either 2NP or 4NP to the corresponding nitrophenoxyl radical [2]. In contrast, photonitration is enabled by the fact that nitrophenols absorb UV-Vis radiation, which induces their photolysis to form the corresponding nitrophenoxyl radicals as the primary reaction intermediates [58]. These radicals can then react with photogenerated ^•^NO_2_ to finally produce 24DNP (Scheme 9).

Interestingly, the formation rate of 24DNP by irradiated nitrite shows an inflection point at pH ~ 3.5, despite the fact that nitrophenols do not react with HNO_2_ directly [2]. The issue is that HNO_2_ is a more effective photochemical and/or thermal source of ^•^NO_2_ (reactions 19–21) compared to NO_2_^−^, and photogenerated ^•^NO_2_ can then react with the nitrophenoxyl radicals produced by irradiation of nitrophenols.

### 2.6. Photonitration by HOONO/H_2_OONO^+^: The Case of Naphthalene/NO_3_^−^/hν

Naphthalene is a relatively electron-poor aromatic compound that, similarly to nitrophenols, cannot undergo nitration by either ^•^NO_2_ or HNO_2_. However, unlike nitrophenols, naphthalene does not absorb environmental UV radiation, thus, a photolysis/nitration pathway similar to that reported in Scheme 9 cannot be operational in this case. By completely excluding the main nitrating agents produced by nitrate photolysis (^•^NO_2_, HNO_2_), naphthalene could be a good substrate for photonitration induced by the nitrating species produced in a lesser amount (H_2_OONO^+^ and HOONO). The available experimental evidence suggests that naphthalene can actually undergo nitration by H_2_OONO^+^ to mainly yield 1-nitronaphthalene, and by HOONO to mainly produce 2-nitronaphthalene [40]. Because the two nitronaphthalene isomers are produced to a large extent by different nitrating species, one could expect the pH trends of the respective formation rates to be different. Indeed, as suggested by Figure 2, the formation rate of 1-nitronaphthalene was directly proportional to [H^+^], while that of 2-nitronaphthalene was dependent on pH to a much lesser extent in the tested acidic range [40]. For naphthalene, the involvement of H_2_OONO^+^ and HOONO in photonitration could be highlighted because of the lack of reactivity with HNO_2_ and ^•^NO_2_.

Photonitration by HOONO in the presence of NO_3_^−^/hν has also been reported for benzene, which is another relatively electron-poor substrate that does not react with either ^•^NO_2_ or HNO_2_. The formation rate of nitrobenzene did not show a significant pH trend in the acidic region, as expected for a process involving HOONO (see Figure 2) [59].

### 2.7. Nitrosation/Oxidation by Photogenerated HNO_2_: The Cases of Catechol and 1-Naphthol with NO_3_^−^/hν

Nitration of both catechol and 1-naphthol (1-hydroxynaphthalene) was observed upon irradiation in the presence of nitrate, with respective production of 4-nitrocatechol and of 2-nitro-1-naphthol [43]. The formation rates of both nitroaromatics under irradiation conditions showed a maximum around pH 3. Both catechol and 1-naphthol react with HNO_2_ in the dark to yield the mentioned nitroderivatives, but the pH trend of the two nitroaromatic compounds is quite peculiar. In both cases one observes initial formation rates with an inflection point around pH 3.5, as expected by a direct involvement of HNO_2_ in the nitration process (Figure 2). However, when focusing on the concentration values of the nitroderivatives reached after 30–40 min reaction time, one observes a pH 3 maximum that is very similar to the pH trend observed in the photonitration process. The maximum around pH 3, together with the fact that this phenomenon is observed only after a certain time lapse, suggests that HNO_2_ triggers a nitrosation/oxidation process in addition to the direct nitration (Figure 2). Coherently, nitrosoderivatives of both catechol and 1-naphthol have been detected in the presence of HNO_2_ in the dark [43].

Overall, the mentioned photonitration process observed in the presence of irradiated nitrate with catechol and 1-naphthol can be accounted for in the hypothesis that the nitrating agent is HNO_2_, operating mainly through a nitrosation/oxidation pathway [43]. Oxidation of the nitrosoderivatives is likely to be faster in the presence of nitrate under irradiation compared to HNO_2_ in the dark, considering that in the former conditions one also has the photogeneration of ^•^OH, which is a very strong oxidising agent [60,61]. A nitrosation/oxidation process is also believed to be responsible for the considerable formation of 4-nitrophenol upon irradiation of phenol and nitrite in basic solution (optimal pH for this process is in the range 9–10, and nitrosation of phenol to 4-nitrosophenol is likely induced by ^•^NO_2_ + ^•^NO instead of HNO_2_) [62].

### 2.8. Proposal of a Protocol for the Study of Photonitration Pathways

As mentioned in previous sections, photonitration is actually a very complex process that stems from the generation of a wide range of different nitrating agents, which differ in the produced amount and intrinsic reactivity. Therefore, the actual production of the nitroderivatives results from the combination of substrate activation to the nitration process, and from the availability of the nitrating agent. However, as a rule of thumb, the electron-rich compounds are more likely to undergo nitration mostly by ^•^NO_2_ and HNO_2_ (HOONO and H_2_OONO^+^ are also reactive [54,63], but they occur in a lesser amount). In contrast, electron-poor substrates that are unreactive towards ^•^NO_2_/HNO_2_ can only be nitrated by HOONO/H_2_OONO^+^. A partial exception to this rule is represented by electron-poor phenols absorbing UV-Vis radiation, such as the nitrophenols. These compounds are unreactive towards ^•^NO_2_, but radiation absorption yields the corresponding phenoxyl radicals [64] that easily produce nitrophenols when reacting with ^•^NO_2_ itself.

In the case of a compound for which reactivity is still unknown, it might be very useful to proceed to the study of the photonitration pathway in a systematic way. Based on previous knowledge, which has been gathered with a range of compounds showing different reactivity towards photonitration, the following actions are foreseen:

1. In neutral solution, there is a real chance for ^•^NO_2_ to be involved in photonitration (especially with electron-rich substrates). Addition of ^•^OH scavengers such as alcohols may help supporting the possible involvement of ^•^NO_2_—indeed, photonitration by nitrate should be enhanced upon addition of the scavengers, if it involves ^•^NO_2_ (solvent-cage effect, see Scheme 1). In contrast, ^•^OH scavengers should inhibit photonitration by nitrite (^•^OH is needed to oxidise NO_2_^−^ to ^•^NO_2_, see Scheme 2);
2. The effect of pH is key to differentiate between different reactive species, especially in the case of nitrate irradiation. Therefore, photonitration rates should be studied as a function of pH with irradiated nitrate, comparing the results with those reported in Figure 2. In several cases, such a comparison will give a clue as to which reactive species is mostly involved in the process;
3. If the pH trend of photonitration by NO_3_^−^/UV suggests the possible involvement of HNO_2_, it is advisable to directly study the reactivity of the substrate with HNO_2_ in the dark (HNO_2_ is produced by acidifying an NO_2_^−^ solution). Direct nitration by HNO_2_ is studied by taking into account the initial formation rates of the nitroderivatives, while the nitrosation/oxidation process is highlighted by quantifying the concentration values reached by the nitroderivatives after a certain amount of time (e.g., 30 to 60 min reaction time in the dark). Note that the oxidation of the nitrosoderivatives might be faster with NO_3_^−^ under irradiation than with HNO_2_ in the dark;
4. If, in contrast, the pH trend of photonitration suggests the involvement of HOONO or H_2_OONO^+^, one should study the dark reactivity of these species. HOONO can be produced in the dark by the reaction of H_2_O_2_ + HNO_2_ [54]. An important issue is that the dark formation of HOONO involves H_3_O_2_^+^ + HNO_2_, thus the pH trend of nitroderivatives formed by HOONO in the dark would show an inflection point at pH ~ 1.5 [54], which is not expected to be seen in the corresponding process taking place under irradiation. In contrast, the involvement of H_2_OONO^+^ in dark nitration would still produce nitroderivative formation rates that are ∝ [H^+^].

By considering together the results of scavenger addition, pH trend and dark control experiments, one should gather enough information to understand which photonitration processes are prevailing for a given substrate under different conditions.

### 2.9. Possible Occurrence of Photonitration in Natural Waters

Evidence of the possible occurrence of photonitration processes in the natural environment has been obtained in the case of natural surface waters, although biological nitration pathways cannot be ruled out. In particular, it appears that the environmental conditions of the Rhône delta lagoons (shallow and sunlit waters, elevated concentrations of nitrate and nitrite) are particularly favourable to the transformation of phenolic compounds, arising from the environmental dissipation of phenoxyacid herbicides, into the corresponding nitroderivatives [31,65,66,67,68,69]:

- 2,4-Dichlorophenoxyacetic acid → 2,4-dichlorophenol → 2,4-dichloro-6-nitrophenol
- MCPA → 4-chloro-2-methylphenol → 4-chloro-2-methyl-6-nitrophenol
- Dichlorprop → 4-chlorophenol → 2-nitro-4-chlorophenol
  where MCPA = 4-chloro-2-methylphenoxyacetic acid, and dichlorprop = 2-(2,4-dichlorophenoxy)propanoic acid.

**Scheme 10:**
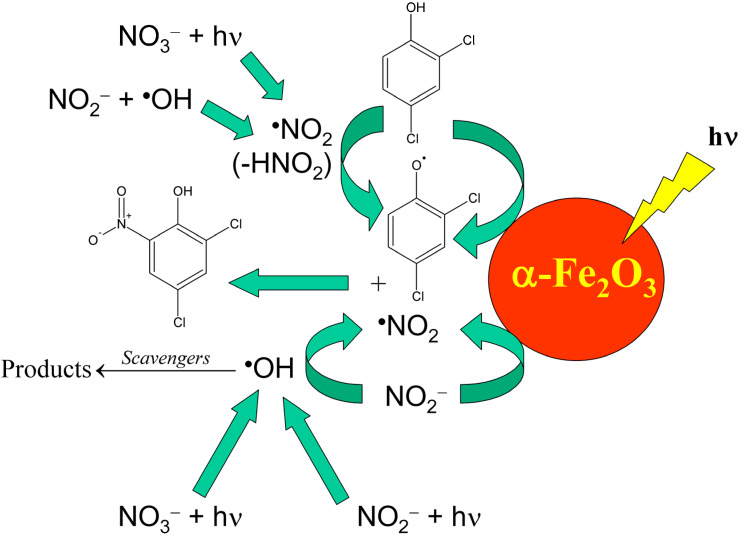
Environmental reaction pathways that could account for the photochemical nitration of 2,4-dichlorophenol into 2,4-dichloro-6-nitrophenol.

It should be remarked that natural waters are indeed much more complex compared to simplified laboratory systems, thus interaction between different components is highly likely. For instance, nitrate and nitrite occur together in the Rhône-delta lagoon water, and the processes they may trigger differ from the mere sum of the separate photoreactions. In particular, the oxidation of NO_2_^−^ to ^•^NO_2_ can be carried out by the ^•^OH radicals produced by both nitrate and nitrite photolysis [70]. Moreover, surface waters contain additional components that can affect the process. For example, iron oxides such as hematite (*α*-Fe_2_O_3_) have been shown to remarkably favour the photonitration both of phenol [71] and of the chlorinated phenols that undergo nitration in the Rhône delta lagoons [31,67]. Further, the oxidation of NO_2_^−^ to ^•^NO_2_ by ^•^OH can be inhibited by a number of ^•^OH scavengers that occur in natural waters, such as natural organic matter and inorganic carbon. That said, Scheme 10 shows a summary of the possible reaction pathways that might account for the photonitration of 2,4-dichlorophenol into 2,4-dichloro-6-nitrophenol in lagoon waters. Similar reaction pathways are likely to be operational for the other compounds.

## 3. Conclusions

The irradiation of nitrate and nitrite ions under environmentally significant UV radiation or sunlight yields a number of nitrating agents (^•^NO_2_, HNO_2_, HOONO, H_2_OONO^+^), which account for the formation of nitroderivatives of a large number of aromatic compounds. This so-called photonitration process can proceed differently depending on the aromatic substrate under consideration, its reactivity towards the different nitrating agents (which depends on its electron-rich or electron-poor nature) and, in some cases, the substrate’s ability to absorb radiation and undergo photolysis as a consequence. Photonitration is usually favoured at acidic pH, albeit with different trends, depending on the involvement of HNO_2_, HOONO and/or H_2_OONO^+^ in the process. Indeed, the study of the pH trend of nitroderivative formation is one of the most effective ways to understand the details of the process. Additionally, ^•^OH scavengers such as alcohols can enhance the formation of the nitroderivatives in the presence of irradiated nitrate, and inhibit it with irradiated nitrite. The reason is, on the one side, the fact that ^•^OH scavengers at large enough concentrations can interfere with the geminate recombination processes of the radical fragments (O^•−^ and ^•^NO_2_) that occur in the solvent cage soon after nitrate photolysis, thereby enhancing ^•^NO_2_ formation. In the case of nitrite, ^•^OH is essential for the oxidation of NO_2_^−^ to ^•^NO_2_, which is understandably inhibited by scavenger addition.

There is evidence that photonitration may be operational in the shallow lagoon water of the Rhône river delta (S. France), involving chlorophenols that are produced as transformation intermediates of chlorophenoxyacid herbicides, and that are transformed into the corresponding nitroderivatives. In such a system, the different photoactive components (nitrate, nitrite, Fe oxide colloids) may interact among themselves, with the substrates, with the nitrating agents and with the natural ^•^OH scavengers (organic matter, inorganic carbon), thereby providing a process that is unavoidably more complex than those occurring in laboratory solutions under irradiation.

The occurrence of nitrate and nitrite in water and wastewater opens up the additional possibility for nitroaromatic compounds to be formed also in the framework of photochemical processes for water treatment, where radiation is often provided by UV lamps and where the aromatic substrates may occur either as contaminants, or as components of the natural organic matter. In some cases, nitrite may even occur together with strong oxidising agents intended for pollutant degradation, yielding nitroderivatives upon oxidation to ^•^NO_2_ [72,73,74,75].

Finally, if the photonitration process was also operational in atmospheric aerosols, it might contribute to the formation of a range of compounds having phytotoxic and mutagenic properties, and would affect the aerosol’s ability to absorb sunlight. The latter phenomenon would influence the earth’s radiation budget, with potential climatic implications.

## Data Availability

Data availability is not applicable to this review paper.

## Sample Availability

Sample availability is not applicable to this review paper.
